# Validation of the Chinese version of muscularity attitudes questionnaire in college students

**DOI:** 10.3389/fpsyg.2026.1732786

**Published:** 2026-05-29

**Authors:** Zitong Xu, Songpeng Su, Ziqi Xu, Yunfei Tao, Xing Zhang, Mingyue Yin, Hansen Li

**Affiliations:** 1 School of Physical Education, Wuhan University of Technology, Wuhan, China; 2 School of Athletic Training, Guangzhou Sport University, Guangzhou, China; 3 Department of Physical Education, Sun Yat-sen University, Guangzhou, China; 4 School of Sports Training, Chengdu Sport University, Chengdu, Sichuan, China; 5 Department of Physical Education and Sport, Faculty of Sport Sciences, University of Granada, Granada, Spain; 6 The Mary MacKillop Institute for Health Research, Australian Catholic University, Melbourne, VIC, Australia; 7 School of Physical Education, Sichuan Agricultural University, Ya'an, China; 8 College of Physical Education, Southwest University, Chongqing, China

**Keywords:** body image, exercise, fitness, instrument, public health

## Abstract

The Drive for Muscularity Attitudes Questionnaire (DMAQ) is a brief tool for assessing muscularity-related attitudes. This study translated the DMAQ into Chinese and evaluated its reliability and validity separately in males and females. A total of 360 Chinese college students completed the questionnaire, and 69 completed a retest one week later. Item analysis, exploratory factor analysis, confirmatory factor analysis, convergent validity analysis, internal consistency analysis, and test-retest reliability analysis were conducted. Items 1 and 6, both reverse-worded items, showed weak factor loadings and were removed. The revised 6-item DMAQ showed acceptable factor loadings in both sexes, although one item was slightly below 0.50 in males. Most model fit indices supported the one-factor structure, but RMSEA values were above the conventional threshold. The revised scale showed good internal consistency in males and females, with Cronbach's alpha values of 0.874 and 0.917, respectively. Test-retest reliability was moderate overall (ICC = 0.625, 95% CI: 0.304 to 0.792). Convergent validity was partially supported, with stronger correlations observed with the Drive for Muscularity Scale than with other related variables. The revised 6-item Chinese DMAQ showed acceptable reliability and generally supportive structural validity among Chinese college students. However, further validation is needed because of the removal of reverse-worded items, elevated RMSEA values, and partial convergent validity evidence.

## 1 Introduction

In recent years, with globalization and sociocultural shifts, body image ideals have become increasingly salient worldwide, particularly the ideal of male muscularity. Many men and adolescent boys associate a muscular physique with confidence, strength, and social desirability (Grogan and Richards, 2002). Under the influence of social media, the pursuit of muscularity may be further intensified, as repeated exposure to idealized and often unrealistic body-related content can foster dissatisfaction with one's own body or level of muscularity (Bocci Benucci et al., 2024; Lowe-Calverley and Grieve, 2021; Tiggemann and Anderberg, 2020). Such concerns are important because excessive emphasis on muscularity has been linked to problematic outcomes, including excessive exercise (Specter and Wiss, 2014), disordered eating (Glazer et al., 2021), and substance abuse (Kanayama et al., 2012).

To study these issues systematically, several psychological instruments have been developed to assess drive for muscularity and related constructs. One of the most widely used measures is the Drive for Muscularity Scale (DMS) (McCreary and Sasse, 2000). Subsequent psychometric work identified two dimensions within the DMS, namely muscularity attitudes and muscularity behaviors (McCreary et al., 2004). However, McCreary et al. (2004) also noted that although the total score may be applied in both men and women, the attitudinal and behavioral subscales are more appropriate for male samples. These findings suggest that muscularity-related constructs can be measured in different ways and that instrument selection should depend on the specific conceptual focus of a study.

Although much of the early research on drive for muscularity was conducted in Western contexts, muscularity-related concerns are also evident in China. Earlier cross-cultural work suggested that Chinese-speaking men may historically have reported lower levels of muscularity-related body dissatisfaction than Western men (Yang et al., 2005). However, later studies in Chinese samples have shown that such concerns are clearly present. Among Chinese adolescents, body dissatisfaction, body-change behaviors, and sociocultural body-image pressures have been documented, with boys reporting pressure to increase muscularity and engaging in muscle-gain behaviors (Xu et al., 2010). Research in Chinese adults has further shown that appearance pressures and muscularity internalization are positively associated with drive for muscularity in both men and women (Barnhart et al., 2022), while recent work has linked muscularity teasing to eating- and body-image disturbances (He et al., 2024). Longitudinal evidence also suggests that muscularity dissatisfaction forms part of the contemporary body-image landscape in China (Barnhart et al., 2024; Xiao et al., 2025). Given the rapid pace of digitalization and the pervasive influence of social media, these concerns may become even more salient in Chinese populations (Lang and Ye, 2024; Yu et al., 2023).

In response to this growing research interest, some muscularity-related instruments have already been developed or adapted for Chinese populations, including the Muscularity Bias Internalization Scale (He et al., 2023a, 2022), the Muscularity-Oriented Eating Test (He et al., 2023b), the Muscle Appearance Satisfaction Scale (Jin et al., 2015), and the Chinese version of the DMS (He et al., 2021). However, instruments specifically assessing muscularity-related attitudes remain relatively limited in Chinese populations. In emerging research areas, the availability of multiple instruments can be valuable because different measures may vary in conceptual emphasis, support triangulation across tools, and facilitate more robust comparisons across studies.

Against this background, the Drive for Muscularity Attitudes Questionnaire (DMAQ) is a relevant instrument for further cross-cultural examination. The DMAQ was originally developed in Canadian men to assess attitudes toward muscularity (Morrison et al., 2004). In the original Canadian validation work, an initial pool of 41 items was reduced to eight items that loaded on a single factor, with acceptable internal consistency (α = 0.84) and evidence of construct validity. Further Canadian evidence also supported the unidimensional structure and internal consistency of the scale (α = 0.82) (Morrison and Harriman, 2005). Subsequent work in British male undergraduate students reported acceptable internal consistency and temporal stability, with Cronbach's alpha ranging from 0.70 to 0.90 and a 14-days intraclass correlation coefficient of approximately 0.78 (Tod et al., 2012). Cross-cultural evidence from Irish men also supported the psychometric soundness of the DMAQ, with confirmatory factor analysis indicating acceptable fit for a one-factor model and evidence of convergent and discriminant validity (Ryan and Morrison, 2014). In addition, the Persian version of the DMAQ has been evaluated among Iranian athletes, where exploratory and confirmatory factor analyses generally supported a one-factor structure, although the internal consistency estimate was lower than that reported in most Western samples (Shahhosseni et al., 2018). Collectively, these findings suggest that the DMAQ has shown broadly acceptable psychometric performance across several cultural and population contexts, but they also indicate that its performance may vary across samples and that most prior validation evidence has been obtained from male participants. Therefore, further validation in Chinese populations, and examination of the scale separately by sex, remains necessary.

The DMAQ was selected for the present study not simply because it is brief, but because it is conceptually well aligned with our specific aim of assessing muscularity-related attitudes rather than broader muscularity-related tendencies. Although the DMS has been widely used, it includes both attitudinal and behavioral components in men (McCreary et al., 2004), whereas the DMAQ focuses more specifically on the attitudinal aspect of muscularity. Thus, the rationale for introducing the DMAQ is not that existing instruments are inadequate, but that an additional instrument with a more specific attitudinal focus may broaden the measurement toolkit available for Chinese research on muscularity-related concerns. This can, in turn, give researchers greater flexibility to choose instruments according to their study needs, or even to use multiple measures simultaneously, thereby reducing the risk that a study may fail or that an entire variable may need to be discarded because of the poor performance of a single instrument. Its brevity and unidimensional structure may also provide practical advantages in large-scale or time-constrained studies, but these should be regarded as secondary strengths rather than the primary reason for its selection.

Therefore, the present study aimed to translate the DMAQ into Chinese and evaluate its psychometric properties in Chinese college students. Because the DMAQ was originally developed in males and perceptions of muscularity may differ by sex, the analyses were conducted separately for male and female participants.

## 2 Materials and methods

### 2.1 Study design and participants

This study recruited a sample of college students, with recruitment conducted in public areas on university campuses. The recruitment process began on November 1, 2024, and lasted for two weeks. Research team members approached potential participants and guided them in completing the questionnaire on their smartphones in real time. Eligibility criteria included being an enrolled college student and having basic physical capabilities (i.e., being able to participate in standard physical education courses). These criteria were selected because the present study aimed to validate the Chinese DMAQ in a general, non-clinical college population. Restricting the sample to enrolled college students helped align the target population with the study aim, whereas the requirement of basic physical capabilities served as a pragmatic indicator that the questionnaire items related to muscularity, exercise, and body-related attitudes would be applicable within a typical student context.

Before completing the questionnaire, all participants were fully informed about the purpose of the study and the intended use of their data. Each participant provided written informed consent, ensuring that participation was entirely voluntary. A total of 360 students voluntarily completed the questionnaire. Among them, 69 participants completed the questionnaire again one week later to evaluate the test-retest reliability of the measurement tool. As part of data screening, we examined the response distributions for potentially invalid patterns, including invariant responding across the questionnaire, and no such cases were identified. The design and conduct of this study were supervised and approved by the Ethics Committee of the College of Physical Education, Southwest University (code: 2023307010).

### 2.2 Measurement tools

#### 2.2.1 The drive for muscularity attitudes questionnaire

The Drive for Muscularity Attitudes Questionnaire (DMAQ) is a psychological measurement tool originally developed by Morrison et al. (2004) in a Canadian male sample to assess individuals' subjective drive for muscularity. The questionnaire consists of 8 items, each rated on a 5-point Likert scale, ranging from “strongly disagree” to “strongly agree.” The tool has a unidimensional structure. Items 1 and 6 of the original DMAQ are reverse-worded items. These items were reverse-coded prior to all psychometric analyses.

In this study, we used the translation procedure adopted by Cheung et al. (2020). Specifically, two groups of translators, each consisting of two members, independently translated the scale forwards and backwards. After several rounds of discussions and revisions, we finalized the translated version as in Supplementary Table S1.

#### 2.2.2 The drive for muscularity scale

The Drive for Muscularity Scale (DMS) is a 15-item measurement tool divided into two dimensions: muscularity attitudes (7 items) and muscularity behaviors (8 items). Each item is rated on a 6-point Likert scale, ranging from “never” to “always.” Research has shown that the DMS demonstrates good structural validity and measurement performance in male populations, though its performance in female populations is relatively weaker (McCreary et al., 2004). This scale showed acceptable overall internal consistency in our full sample (Cronbach's α = 0.92).

#### 2.2.3 The muscularity bias internalization scale

The Muscularity Bias Internalization Scale (MBIS) is a measurement tool developed for Chinese males to assess the degree to which individuals internalize muscularity-related stereotypes and experience negative self-evaluations due to not achieving the ideal muscular physique (He et al., 2022). The scale consists of 14 items and has a three-dimensional structure, including: Personal Value attached to Muscularity (PVM), Perceived Impact of Muscularity (PIM), and Definition and Appearance of Muscularity (DAM). This scale showed acceptable overall internal consistency in our full sample (Cronbach's α = 0.94).

#### 2.2.4 Physical activity rating scale

Physical activity (PA) was assessed using the Physical Activity Rating Scale (PARS) (Liang, 1994). This scale comprises three items measuring physical activity intensity, duration, and frequency. Each item is rated on a 5-point Likert-type scale. For instance, the response options for PA frequency range from “less than 1 time/month (1 point)” to “every day (5 points),” with intermediate options such as “2 to 3 times/month (2 points),” “1 to 2 times/week (3 points),” and “3 to 5 times/week (4 points).” General physical activity levels were calculated by multiplying the intensity score, the duration score minus one, and the frequency score. This scale showed acceptable overall internal consistency in our full sample (Cronbach's α = 0.71).

#### 2.2.5 Demographic variables

We also collected demographic information, including sex, age, and academic year, to characterize the sample. These variables were included as background sociodemographic indicators rather than as primary validation variables. This decision was based on prior literature suggesting that body image and related concerns may vary across demographic contexts (Mak et al., 2013). The categorization methods for these variables are presented in the Results section.

### 2.3 Statistical analysis

#### 2.3.1 Common method bias detection

Because all questionnaire data were collected using self-report measures, we examined the potential influence of common method bias using Harman's single-factor test (Zhou and Long, 2004). In this test, all questionnaire items were entered into an unrotated exploratory factor analysis. Common method bias was considered unlikely to be a serious concern if the first unrotated factor accounted for less than 40% of the total variance.

#### 2.3.2 Item analysis

Item analysis was conducted to examine the preliminary performance of each DMAQ item. Specifically, we calculated the corrected item-total correlation (based on Spearman correlation) for each item separately in male and female participants.

#### 2.3.3 Validity

Before conducting the confirmatory factor analysis (CFA), we performed an exploratory factor analysis (EFA). We randomly split the sample at a 1:1 ratio based on randomly generated numbers. This resulted in a sample of 164 participants for EFA, including 90 males, and a sample of 196 participants for CFA, including 108 males. Prior to factor extraction, the suitability of the data for factor analysis was assessed using the Kaiser-Meyer-Olkin (KMO) measure and Bartlett's test of sphericity, with KMO > 0.70 and a significant Bartlett's test (*p* < 0.05) considered acceptable. Factors were then extracted using principal component analysis with varimax rotation.

Thereafter, we used CFA to evaluate structural validity. The analysis was conducted using Structural Equation Modeling (SEM) with Maximum Likelihood (ML) estimation. To detect potential multicollinearity issues among the variables in the model, we calculated the Variance Inflation Factor (VIF). We ensured that the VIF values for all variables were below 5.0 (Rogerson, 2019), thereby mitigating the risk of multicollinearity.

According to the guidelines of the COnsensus-based Standards for the selection of health Measurement INstruments (COSMIN), the sample size should be at least seven times the number of items in the scale when validating structural validity (Mokkink et al., 2019; Prinsen et al., 2018). Additionally, based on previous experiences with SEM, a sample-to-parameter ratio of 10:1 is considered ideal (Bagozzi and Yi, 2012). Therefore, our sample size meets the analytical requirements.

Since the variables did not fully meet the multivariate normality assumption, we primarily used the Bollene-Stine statistic to evaluate model fit (Bollen and Stine, 1992). This statistic was generated through 10,000 bootstrap iterations, with a *p*-value of Bollen-Stine statistic > 0.05 indicating a good model fit. Additionally, we referred to the following fit indices and their acceptable thresholds: Standardized Root Mean Square Residual (SRMR) < 0.08; Normed Fit Index (NFI) > 0.90; Tucker-Lewis Index (TLI) > 0.90; Comparative Fit Index (CFI) > 0.90; and Root Mean Square Error of Approximation (RMSEA) < 0.08 (Schermelleh-Engel et al., 2003).

Furthermore, we required the standardized factor loadings to be greater than 0.5, the Average Variance Extracted (AVE) to exceed 0.5, and the Construct Reliability (CR) to be higher than 0.6 to ensure the measurement quality of the model (Ahmad et al., 2016; Cheung and Wang, 2017).

Convergent validity refers to how closely a test is related to other tests that measure the same (or similar) constructs (Chin and Yao, 2024). In this study, we explored the relationship between the DMAQ and several similar psychological measurement tools, as well as exercise levels, through Spearman correlation analysis to provide evidence for convergent validity.

We interpreted the correlation coefficients based on the following intervals: 0–0.10: no significant correlation; 0.11–0.39: weak correlation; 0.40–0.69: moderate correlation; 0.70–0.89: strong correlation; 0.90–1.00: very strong correlation (Schober et al., 2018).

#### 2.3.4 Reliability

We used Cronbach's alpha coefficient to assess the internal consistency of the items in each dimension of the scale. Typically, a Cronbach's alpha value greater than 0.7 is considered acceptable for internal consistency (Taber, 2018).

Test-retest reliability refers to the consistency of responses when repeated assessments are made within a stable population (Fayers and Machin, 2013). To assess the test-retest reliability of the scale, we used a two-way mixed effects model based on absolute agreement and single measures to calculate the Intraclass Correlation Coefficient (ICC) (Qin et al., 2019). The interpretation of ICC values is as follows: ICC ≥ 0.75 indicates excellent/high reproducibility; ICC between 0.4 and 0.75 indicates moderate to good reproducibility; and ICC < 0.4 indicates poor or low reproducibility (Ageberg et al., 2007).

Prior to the ICC analysis, we examined several key conditions for test-retest reliability. No outliers were detected using the interquartile range method. In addition, the score distributions at the two time points did not show obvious non-normality, as indicated by acceptable skewness (−0.62 and 0.05) and kurtosis (−0.61 and 1.16) values (Kim, 2013). Nevertheless, a paired-samples *T* test revealed a statistically significant systematic difference between the two measurements, with the mean DMAQ score decreasing from 25.26 at baseline to 22.95 at retest (*p* < 0.01), suggesting the presence of some systematic bias over time.

We estimated the required sample size using an ICC-based sample size calculation (Arifin, 2018). The following parameters were specified: minimum acceptable ICC = 0.50, expected ICC = 0.80, significance level = 0.05, power = 0.80, two repeated measurements, and an anticipated dropout rate of 80%. Based on these assumptions, the required sample size was estimated to be 140 participants at the first assessment. All participants who completed the baseline survey were invited to complete the DMAQ again after one week; 69 of 360 participants completed the second assessment, corresponding to an attrition rate of 80.83%. The COSMIN guidance considers a sample size of 50–99 participants to be adequate and < 30 to be inadequate for reliability analyses (Mokkink et al., 2019). Therefore, the final retest sample of 69 participants was considered acceptable for the overall test-retest reliability analysis, although the relatively low follow-up rate should be acknowledged and the sex-stratified ICC estimates interpreted with caution.

## 3 Results

### 3.1 Common method bias

Harman's single-factor test showed that the first unrotated factor accounted for 15.89% of the total variance, which was below the commonly used threshold of 40%. Therefore, common method bias was unlikely to seriously affect the present findings.

### 3.2 Descriptive statistics

The sample comprised 360 college students, including 198 males (55.0%) and 162 females (45.0%), with a mean age of 19.39 years (SD = 1.56). Most participants were first- or second-year students (40.3% and 35.0%, respectively), whereas 11.9% were third-year students, 9.4% were fourth-year students, and 3.3% were master's students. More detailed participant characteristics are shown in Supplementary Table S2. Overall, the item means, standard deviations, skewness, and kurtosis values suggested that the items showed acceptable distributional characteristics for subsequent psychometric analyses.

### 3.3 Item analysis

The corrected item-total correlations are shown in Table 1. In the male sample, the corrected item-total correlations ranged from 0.387 to 0.719, and all correlations were statistically significant (*p* < 0.01). In the female sample, the values ranged from 0.409 to 0.802, with all correlations also reaching statistical significance (*p* < 0.01). Notably, Item 1 and 6 showed relatively lower corrected item-total correlation values in both males and females. Both items were reverse-worded items, suggesting that their performance should be further considered in the subsequent factor analyses.

**Table 1:** The corrected item-total correlation.

Item	Male	Female
1	0.387^**^	0.531^**^
2	0.641^**^	0.621^**^
3	0.667^**^	0.743^**^
4	0.694^**^	0.774^**^
5	0.715^**^	0.802^**^
6	0.544^**^	0.409^**^
7	0.719^**^	0.720^**^
8	0.621^**^	0.734^**^

^**^, *p* < 0.01.

### 3.4 Exploratory factor analysis

Exploratory factor analysis indicated that the data were suitable for factor analysis in both males and females, as shown by adequate KMO values and significant Bartlett's tests of sphericity. In males, the KMO value was 0.856 and Bartlett's test was significant (*p* < 0.001). The initial EFA suggested a two-factor solution, with the two reverse-worded items loading on one factor that accounted for only 16.73 % of the variance, whereas the remaining items loaded on another factor explaining 50.80% of the variance. Given that the smaller factor explained relatively little variance, lacked a clear theoretical interpretation, and consisted of only two items, we considered it more likely to reflect a wording- or method-related effect rather than a stable substantive dimension. In females, the KMO value was 0.828 and Bartlett's test was significant (*p* < 0.001). The EFA suggested a one-factor solution, accounting for 61.37% of the variance. Taken together, the EFA results suggested that the reverse-worded items may have introduced a wording-related factor, particularly in males.

### 3.5 Confirmatory factor analysis

Consistent with the exploratory findings, confirmatory factor analysis of the original 8-item DMAQ showed that Items 1 and 6, both of which were reverse-worded and reverse-coded before analysis, had standardized factor loadings below the generally acceptable threshold of 0.50 in both males and females (Supplementary Figure S1). These two items were therefore removed. After removing Items 1 and 6, the remaining six items demonstrated acceptable standardized factor loadings in both sexes (Supplementary Figure S2). In males, the factor loadings ranged from 0.487 to 0.837 (Table 2). Although Item 2 was slightly below 0.50, it was close to the acceptable threshold and was retained to preserve content coverage. In females, the factor loadings ranged from 0.703 to 0.884, indicating stronger item-level performance. The revised 6-item model showed generally acceptable fit in the male sample, although RMSEA was above the conventional threshold: Bollen-Stine *p* = 0.147, SRMR = 0.050, NFI = 0.921, TLI = 0.914, CFI = 0.948, RMSEA = 0.125, AVE = 0.531, and CR = 0.869. Similar findings were observed in the female sample: Bollen-Stine *p* = 0.058, SRMR = 0.040, NFI = 0.932, TLI = 0.923, CFI = 0.954, RMSEA = 0.148, AVE = 0.668, and CR = 0.923. Overall, most fit indices supported the revised one-factor structure, although the elevated RMSEA values should be interpreted cautiously.

**Table 2:** Factor loading of the DMAQ (with 6 items).

Item	Factor loading	
	Male	Female
2	0.487	0.703
3	0.699	0.813
4	0.837	0.841
5	0.809	0.884
7	0.773	0.842
8	0.711	0.810

### 3.6 Convergent validity

Spearman correlation analyses were conducted to examine the associations between the DMAQ and theoretically related variables (Table 3). In the male sample, the DMAQ total score showed a weak correlation with the MBIS total score and physical activity level, and a moderate correlation with the DMS total score. In the female sample, the DMAQ total score showed a moderate correlation with the MBIS total score, a strong correlation with the DMS total score, and a weak correlation with physical activity level. Overall, these findings provide partial support for the convergent validity of the Chinese DMAQ. The evidence was stronger for convergence with the DMS than with the MBIS or physical activity, particularly in males.

**Table 3:** The correlations of DMAQ and other relevant variables.

Gender	Coefficient	MBIS	DMS	PARS
Male	rho	0.314^**^	0.499^**^	0.289^**^
	*p*	< 0.001	< 0.001	< 0.001
Female	rho	0.630^**^	0.712^**^	0.394^**^
	*p*	< 0.001	< 0.001	< 0.001

^**^, *p* < 0.01. DMAQ, Drive for Muscularity Attitudes Questionnaire; MBIS, Muscularity Bias Internalization Scale; DMS, Drive for Muscularity Scale; PARS, Physical Activity Rating Scale.

### 3.7 Reliability

The internal consistency coefficients of the scales and subscales used in the study were generally acceptable (Table 4), except for PARS, which showed values slightly lower than the recommended threshold (α = 0.674 for males and 0.693 for females). For the DMAQ, the original 8-item version showed acceptable internal consistency in both males (α = 0.811) and females (α = 0.887), whereas the revised 6-item version showed improved internal consistency in both males (α = 0.874) and females (α = 0.917). The MBIS subscales demonstrated good to excellent internal consistency, with Cronbach's α ranging from 0.781 to 0.964 in males and from 0.850 to 0.957 in females. The DMS subscales also showed acceptable internal consistency in both sexes, with α values ranging from 0.821 to 0.825 in males and from 0.828 to 0.866 in females. In the overall sample, the revised DMAQ showed moderate test-retest reliability (ICC = 0.625, 95% CI: 0.304 to 0.792, *p* < 0.001). When stratified by sex, the scale also demonstrated moderate stability in both males and females, with single-measure ICCs of 0.554 (95% CI: 0.264 to 0.754, *p* < 0.001) and 0.725 (95% CI: 0.034 to 0.909, *p* < 0.001), respectively.

**Table 4:** The internal consistency of the used scales.

Scale	Version/ subscale	Male (Cronbach's α)	Female (Cronbach's α)
DMAQ	Original 8-item version	0.811	0.887
DMAQ	Revised 6-item version	0.874	0.917
MBIS	PVM	0.964	0.957
MBIS	PIM	0.876	0.919
MBIS	DAM	0.781	0.850
DMS	Behavioral subscale	0.825	0.828
DMS	Attitudinal subscale	0.821	0.866
PARS	N.A.	0.674	0.693

DMAQ, Drive for Muscularity Attitudes Questionnaire; MBIS, Muscularity Bias Internalization Scale; PVM, Personal Value attached to Muscularity; PIM, Perceived Impact of Muscularity; DAM, Definition and Appearance of Muscularity; DMS, Drive for Muscularity Scale; PARS, Physical Activity Rating Scale.

## 4 Discussion

### 4.1 General discussion

This study translated the Drive for Muscularity Attitudes Questionnaire (DMAQ) into Chinese and systematically assessed its applicability among Chinese college students. The deletion of Items 1 and 6 requires particular consideration, because both items were reverse-worded. Importantly, these items were reverse-coded prior to analysis, yet they still showed low factor loadings in both male and female subsamples. This suggests that their poor performance was unlikely to be explained simply by a coding error. Rather, it is plausible that the reverse-worded format itself introduced additional measurement difficulty. Reverse-worded items may increase cognitive burden and may be more prone to misunderstanding or inconsistent responding, especially in translated instruments. In the present study, these effects may have been amplified by linguistic and cultural factors in the Chinese adaptation, such that the negated wording did not function as smoothly or naturally as the positively worded items. Therefore, the removal of these two items should not be interpreted only as a data-driven simplification, but also as evidence that reverse-worded DMAQ items may require careful retranslation or rewording in Chinese contexts. Future studies should examine this issue more directly, for example through cognitive interviewing, alternative item wording, or independent validation of revised Chinese versions.

The revised DMAQ with 6 items exhibited strong structural validity and reliability in the male sample. The confirmatory factor analysis (CFA) results showed that, after removing the reverse-scored items, most fit indices met acceptable standards (e.g., NFI, CFI, TLI). The internal consistency (Cronbach's alpha) and test-retest reliability (ICC) were within acceptable ranges. Although the RMSEA value exceeded the conventional threshold, it approached the relaxed cutoff of 0.10 adopted in some studies [e.g., Zucoloto et al. (2016)]. Importantly, we refrained from adding residual covariance links to improve model fit based on modification indices, as has been done in some prior studies (Acuña Mora et al., 2022; Andrea et al., 2024). As suggested by others, we should avoid such modifications made only to improve model fitting (Jöreskog et al., 2016). This is also because these modifications are usually data-driven modifications and lack theoretical justification (Perry et al., 2015). By adhering to the original theoretical structure, we prioritized simplicity and theoretical consistency, which may enhance the generalizability of the results.

Our male findings were broadly in line with previous Western DMAQ research, which has supported a one-factor structure among Irish men (Ryan and Morrison, 2014). Notably, the convergent validity patterns appeared to differ between the male and female subsamples. Among males, the DMAQ was more strongly associated with the DMS than with the MBIS, whereas the correlations with the MBIS and physical activity were relatively weak. Therefore, in the male subsample, the convergent validity evidence should be regarded as only partial rather than strong. In females, the stronger association with the DMS and the moderate association with the MBIS provided comparatively more supportive evidence, although the weak correlation with physical activity again suggests that convergence was not uniform across all related variables. Taken together, these findings suggest that the convergent validity of the Chinese DMAQ is more strongly supported by some comparator measures than others, and future studies should include additional theoretically relevant variables to provide more comprehensive validity evidence.

### 4.2 Limitations and future directions

We were unable to retain all items of the questionnaire. Items 1 and 6, which were reverse-coded, were removed due to low factor loadings that failed to meet model fit requirements. This issue has been observed in other scale translation studies as well (Wang et al., 2024). Reverse-coded items can sometimes lead to comprehension biases or inconsistent responses, especially in non-native language settings where cultural and linguistic differences might amplify these challenges. Future tool development or adaptation efforts should therefore not only consider minimizing reverse-worded items, but also examine whether these two items can be rephrased in a more culturally and linguistically natural way in Chinese rather than being permanently omitted.

The study was conducted exclusively among college students, limiting the generalizability of the findings to other age groups or occupational populations. While the DMAQ underwent an adaptation process, the original scale was developed in Western cultural contexts, and its items might not fully capture the unique conceptualization of muscularity within Eastern cultural frameworks.

Although the DMAQ showed moderate test-retest reliability, a statistically significant difference was observed between the two administrations, suggesting some degree of systematic bias over time. Therefore, the evidence for absolute agreement should be interpreted with caution, and future studies should further examine the stability of the scale under more strictly controlled retest conditions.

## 5 Conclusion

The present study translated the DMAQ into Chinese and evaluated its psychometric properties among Chinese college students. The findings suggest that the original 8-item version did not perform optimally in this sample, mainly because the two reverse-worded items showed weak factor loadings despite being reverse-coded before analysis. After removing these two items, the revised 6-item Chinese DMAQ demonstrated good internal consistency, moderate test-retest reliability, and generally acceptable evidence for a one-factor structure in both male and female students. However, the elevated RMSEA values and the only partial support for convergent validity suggest that the evidence should be interpreted cautiously rather than as definitive validation. Overall, the revised 6-item Chinese DMAQ may serve as a brief tool for assessing muscularity-related attitudes in Chinese college student samples, but further validation is needed in more diverse populations and with additional theoretically relevant external criteria.

## Data Availability

The raw data supporting the conclusions of this article will be made available by the authors, without undue reservation.
