# Supplementary material for: Validation of the Chinese version of muscularity attitudes questionnaire in college students
Source: Front Psychol. 2026 May 29;17:1732786. doi: 10.3389/fpsyg.2026.1732786 (PMC13259729; doi:10.3389/fpsyg.2026.1732786)
Supplement: Supplementary file 1 [file Supplementary_file_1.docx]

Supplementary Material

**Table S1.** The Items of DMAQ in English and Chinese

| Item | Original version (in English) | Translated version (in Chinese) |
| --- | --- | --- |
| **1** | **I do not want to become more muscular.** | **我不想变得更有肌肉。** |
| 2 | I wish my legs were more muscular. | 我希望我的腿部更有肌肉。 |
| 3 | When I see a guy who is really muscular, it inspires me to get bigger myself. | 当我看到一个非常有肌肉的人时，我会受到启发，想要让自己也变得更强壮。 |
| 4 | Muscularity is important to me. | 肌肉发达对我很重要。 |
| 5 | I think I need to gain a few pounds of “bulk”(muscle mass). | 我觉得我需要增加几斤的“肌肉量”（肌肉质量）。 |
| **6** | **I do not wish my arms were more muscular.** | **我不希望我的手臂更有肌肉。** |
| 7 | I should work out more to increase muscle mass. | 我应该多锻炼以增加肌肉量。 |
| 8 | I would feel more confident if my lats (back muscles) were bigger. | 如果我的背部肌肉（背阔肌）更大，我会感觉更自信。 |

**Note:** bolded items indicating reverse scoring.

**Table S2.** Participants’ characteristics

| Variable | Category | N (%) | Mean (SD) |
| --- | --- | --- | --- |
| Sex | Male | 198 (55.0%) | - |
|  | Female | 162 (45.0%) | - |
| Age | - | - | 19.39 (1.56) |
| Grade | First year | 145 (40.3%) | - |
|  | Second year | 126 (35.0%) | - |
|  | Third year | 43 (11.9%) | - |
|  | Fourth year | 34 (9.4%) | - |
|  | Master student | 12 (3.3%) | - |


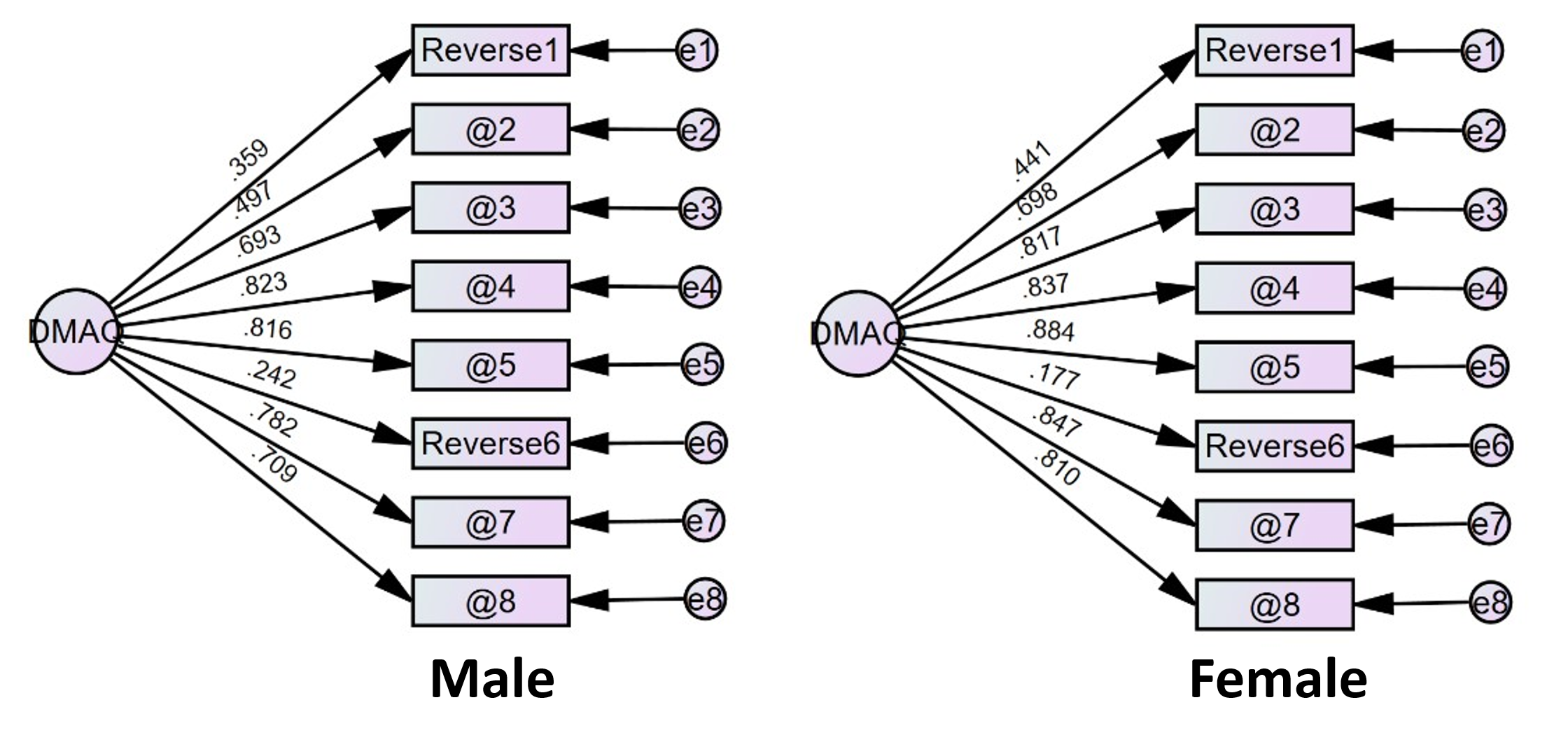


**Figure S1.** The factor loading of the 8-itmes DAMQ (original version)

**
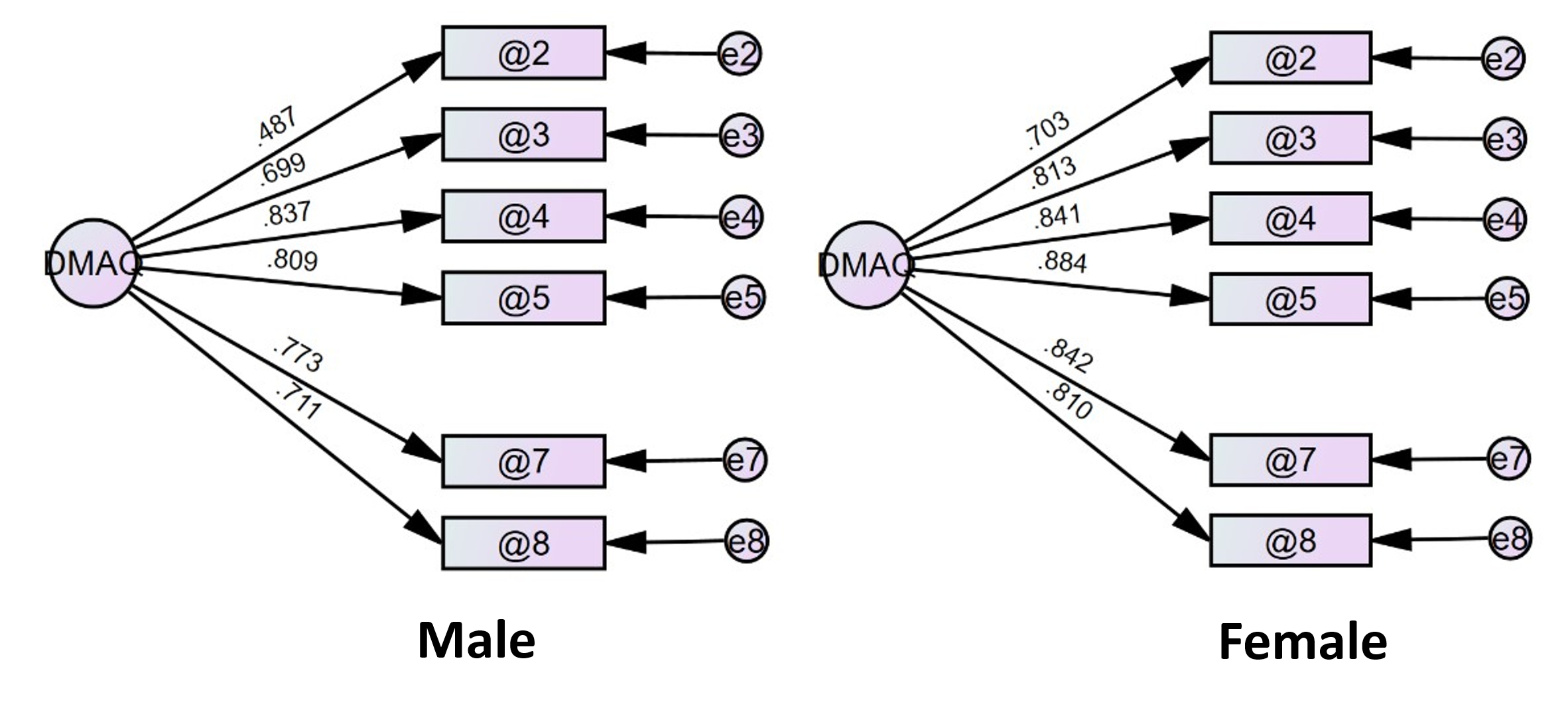
**

**Figure S2.** The factor loading of the 6-itmes DAMQ
